# Salt Intake Among the Iranian Population and Public Attitudes Toward Salt Consumption: National and Subnational Report From STEPS 2021

**DOI:** 10.1002/fsn3.71399

**Published:** 2025-12-30

**Authors:** Nasim Nosratinia, Sina Azadnajafabad, Masoud Masinaei, Ali Golestani, Seyyed‐Hadi Ghamari, Mohsen Abbasi‐Kangevari, Negar Rezaei, Sepehr Khosravi, Shahabeddin Rezaei, Naser Ahmadi, Ameneh Kazemi, Erfan Ghasemi, Yosef Farzi, Mohammad‐Mahdi Rashidi, Moein Yoosefi, Nazila Rezaei, Maryam Nasserinejad, Rosa Haghshenas, Sahar Mohammadi Fateh, Mohammad Keykhaei, Mana Moghimi, Elmira Foroutan Mehr, Azadeh Momen Nia Rankohi, Shirin Djalalinia, Farshad Farzadfar

**Affiliations:** ^1^ Non‐Communicable Diseases Research Center, Endocrinology and Metabolism Population Sciences Institute Tehran University of Medical Sciences Tehran Iran; ^2^ Department of Epidemiology and Biostatistics, School of Public Health Tehran University of Medical Sciences Tehran Iran; ^3^ Human Nutrition Program, Department of Human Sciences The Ohio State University Columbus Ohio USA; ^4^ Department of Mathematics and Statistics Memorial University of Newfoundland St. John's Newfoundland and Labrador Canada; ^5^ Center for Life Course Health Research, Faculty of Medicine University of Oulu Oulu Finland; ^6^ Division of Cardiology, Department of Medicine The Johns Hopkins University School of Medicine Baltimore Maryland USA; ^7^ Development of Research and Technology Center Deputy of Research and Technology Ministry of Health and Medical Education Tehran Iran; ^8^ Endocrinology and Metabolism Research Center, Endocrinology and Metabolism Clinical Sciences Institute Tehran University of Medical Sciences Tehran Iran

**Keywords:** adult, blood pressure, diet, Iran, salt, sodium‐restricted

## Abstract

High salt intake is a significant risk factor for non‐communicable diseases globally. This study aimed to assess salt intake and attitude to salt consumption in Iran in 2021 using STEPS data. Data were analyzed from a population‐based survey with a nationally and sub‐nationally representative sample of Iranian adults. A systematic cluster sampling approach was employed. In total, 17,910 participants were included in the salt intake analyses, and 27,838 participants were included in the attitude and practice analyses. Spot urine samples were collected to estimate 24‐h urine sodium and daily sodium intake. Participants also completed questionnaires, and anthropometric and physical measurements were taken. The mean daily salt intake in Iran in 2021 was 9.71 g/day, with 97.98% of participants exceeding the WHO‐recommended limit of 5 g/day. Salt intake was higher in men than in women (9.95 vs. 9.51 g/day), and higher among rural compared to urban residents (10.02 vs. 9.6 g/day). Married and obese individuals had elevated salt intake. Participants with hypertension consumed more salt than those without (9.83 vs. 9.64 g/day). However, among hypertensive individuals, those aware of their condition consumed less salt. 91.96% of participants thought that excessive dietary salt could cause health problems and 57.93% considered reducing salt intake “very important.” Participants who added salt in their last meal had 1.77 times higher odds of excessive salt intake. Men (OR = 1.48) and those with higher BMI (OR = 1.67 for overweight, 2.26 for obesity) were more likely to exceed recommended salt levels. Our results underscore an urgent public health challenge, as salt intake among Iranian adults is nearly twice the recommended threshold. Habits such as using salt shakers, adding salt during cooking, and consuming salty processed foods were more common among individuals with higher estimated intake. These associations underscore the need for continued national attention to salt reduction efforts.

## Graphical abstract

In 2021, Iranian adults consumed an average of 9.71 g of salt per day, nearly double the WHO‐recommended limit, with 98% exceeding 5 g/day. Intake was higher among men, rural residents, and obese individuals, and was strongly linked to unhealthy behaviors such as adding salt during cooking or meals. Despite widespread awareness of salt's health risks, behavioral change remains limited, underscoring the need for culturally tailored public health interventions and food industry regulation to reduce salt consumption.

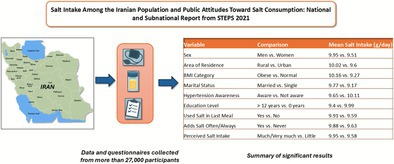

## 1 Introduction

Salt intake and diet high in sodium are major risk factors for non‐communicable diseases (NCDs), particularly high systolic blood pressure, and this notion has been excessively investigated (Chen et al. 2021; Breda et al. 2021). Globally, high intake of salt contributed to 1.89 million deaths and 44.87 million disability‐adjusted life‐years (DALYs) in 2019, which leads to the fact that high salt intake was the leading dietary risk factor for deaths and DALYs worldwide (Chen et al. 2021). World Health Organization (WHO) has estimated that the reduction of salt intake under 5 g/day would prevent 2.5 million deaths annually (World Health Organization 2012). In this regard, the 2030 Agenda for Sustainable Development, adopted by all United Nations Member States in 2015, provides goals targeting a 30% reduction in mean salt intake by 2025, compared with that of 2010 as a baseline year (World Health Assembly 66 2013).

Although the health burden of high salt intake is well established, many countries face gaps in nationally representative, individual‐level data on salt consumption (GBD 2017 Diet Collaborators 2019; GBD 2019 Risk Factors Collaborators 2020). Highlighting the importance of national surveillance services for monitoring NCD risk factors, the WHO recommended the stepwise approach for surveillance (STEPS) as a cardinal framework for periodically evaluating the NCDs status in countries and territories (World Health Organization 2023b).

The first national survey of salt intake in Iran using the STEPS method was done in 2016. The mean salt intake was 9.5 g/day among the Iranian adult population (Rezaei et al. 2018). Iran, as a developing country, faces a major socioeconomic transition during the past century, including population growth, an increase in the number of elderly people, and rapid and unplanned urbanization, which has led to a reduction in the burden of communicable diseases and a shift to the NCDs epidemic in recent decades (Danaei et al. 2019). Nevertheless, the healthcare system has not yet been aligned commensurate with such dramatic changes (GBD 2019 Iran Collaborators 2022). Therefore, intermittently monitoring NCDs situations and their risk factors would empower the policymakers to capture a clearer picture of the situation. This study provides the first follow‐up to the 2016 survey, offering nationally and provincially representative data from 2021 that allow for comparison over time and assessment of policy progress, with the objective of investigating salt intake in the Iranian population along with related attitudes and practices across sociodemographic groups and key NCD risk factors.

## 2 Materials and Methods

### 2.1 Study Design and Participants

In this study, we used the data from the STEPS 2021 and reported the situation of salt intake in the Iranian adult population. The STEPS 2021 study was conducted in three steps: Step 1 involved data collection using a questionnaire, Step 2 included anthropometric measurements, and Step 3 consisted of laboratory measurements. The details about data collection and the method of the study have been explained in detail previously (Djalalinia et al. 2022). To obtain a nationally and sub nationally representative sample, a systematic cluster sampling approach was employed, taking into account the population distribution across provinces and applying relative weighting. The estimated sample size was 28,821, of which 27,874 individuals completed the questionnaire. The study included Iranian adults aged 18 years and older. Individuals with psychological disorders that hindered their ability to complete the questionnaire, those with physical disabilities preventing anthropometric measurements, those for whom laboratory data could not be collected, and pregnant women were excluded. Subsequently, physical measurements were successfully conducted on 27,745 participants, and laboratory samples were obtained from 18,119 individuals. While all adults aged 18 and above were eligible for the initial and second stages of the study, participation in the third stage, which involved laboratory testing, was restricted to individuals aged 25 years and older. Regarding analyses, for salt intake related outcomes we included participants with available data of their estimated salt intake, while for outcomes related to attitude to salt consumption, we included participants without missing values for at least one of the related questions.

### 2.2 Data Collection

In the first step of the STEPS survey, the following data were collected with a questionnaire: demographic features, vegetable and fruit consumption, attitude to salt consumption, past medical history, physical activity, smoking, and family assets. The questionnaire was mainly based on the WHO questionnaire (version 3.2) for STEPS, but with added and modified questions specific for this study (Djalalinia et al. 2022). In the second step, anthropometric measurements, including height, weight, and blood pressure were performed. To ensure data reliability, field staff were trained and certified before data collection, with refresher training conducted periodically. Height was measured using a standard stadiometer, with participants standing upright against a wall so that their heels, buttocks, and the back of their head touched the surface. Body weight was recorded using a calibrated digital scale (Inofit), with participants wearing light clothing and no shoes. Prior to blood pressure assessment, participants rested in a seated position for 15 min. Blood pressure was then measured three times at three‐minute intervals using a standard Beurer sphygmomanometer. The average of the second and third readings was used as the participant's final blood pressure value.

In the third step, laboratory measurements including total serum cholesterol and fasting plasma glucose (FPG) and spot urine samples were collected. All samples were collected in the morning and transferred to the central laboratory of the Non‐Communicable Diseases Research Center considering the optimal condition criteria defined in the protocol (Djalalinia et al. 2022). Participants were instructed to void their first morning urine at home. A random urine sample was then obtained at the study site after fasting blood collection. A total of 17,910 individuals provided spot urine samples and underwent urine sodium measurement. In addition to urine sodium, the kinetic colorimetric assay was applied to assess the spot urine creatinine based on the Jaffe method (Toora and Rajagopal 2002). 24‐h (24‐h) urine sodium estimation in population studies was mainly determined by applying three known equations, namely Kawasaki, Tanaka, and INTERSALT methods (Ma et al. 2017; Brown et al. 2013; Tanaka et al. 2002; Kawasaki et al. 1993). Although the 24‐h urine collection remains the gold standard for assessing sodium intake, it poses logistical challenges and participant burden (Bahadoran et al. 2022). Consequently, the WHO recommends the use of spot urine samples as a practical alternative in population surveys such as the STEPS studies (World Health Organization 2025b). Spot urine sampling is considered acceptable at the population level because, when applied to validated predictive equations, it can provide reliable estimates of mean 24‐h sodium excretion (Conkle and van der Haar 2016).

A systematic review has demonstrated that mean salt intake derived from spot urine samples offers countries a useful indication of population‐level sodium consumption and whether public health interventions are warranted (Huang et al. 2016). However, there is limited evidence supporting the superiority of one predictive equation over another across different populations or contexts (Rhee et al. 2014). Researchers have selected among predictive equations for various reasons—some following validation analyses in their specific populations (Meyer et al. 2019; Webster et al. 2016), while others have chosen based on comparability with previous surveys or alignment with commonly used methods in similar settings (Meyer et al. 2019; Bhattarai et al. 2022; Petersen et al. 2017; Clermont et al. 2023; Holvik et al. 2024; Paterson et al. 2019; Do et al. 2016).

The validity of these methods in estimating daily salt intake in the Iranian population has been investigated previously (Mohammadifard et al. 2020). In the present study, we applied the same approach used in the national STEPS 2016 survey (Rezaei et al. 2018). To choose the most appropriate model among the abovementioned validated methods, 24‐h urine samples were collected from a subsample of 623 participants. Root mean square error was calculated for all equations. The root mean square error for Tanaka, Kawasaki, and INTERSALT equations is presented in Table 1. Finally, due to the lower mean root square error value and no negative values in the confidence intervals, the Tanaka method was chosen for this study. Considering the 10% excretion of sodium through sweating, the value was added to the selected equation results (Baker 2017). The final selected equation was similar to the STEPS 2016 study, ensuring consistency and comparability.

**TABLE 1:** Estimated mean salt intake and 24‐h urine sample.

Method	No. of participants	Mean (SD)	Min	Max	Root mean square error (SD)
Kawasaki	17,910	11.2 (0.00)	1.36	195.19	5.11 (1.39)
Tanaka	17,910	8.82 (0.00)	1.77	81.55	3.83 (1.14)
INTERSALT	17,888	8.14 (0.00)	−1.12	19.08	3.53 (1.06)
24 h urine	623	7.94 (0.2)	1.64	26.59	N/A

### 2.3 Variable Definition

The primary outcomes of this study were high salt intake and attitudes toward salt consumption. Appropriate salt intake was classified based on the WHO recommendations, which define appropriate intake as less than 5 g/day (World Health Organization 2012). Attitudinal data were collected through several questionnaire items, including:

- Whether salt was added to the last lunch or dinner meal (yes/no);
- Frequency of adding salt to food at the table or just before eating (never/rarely/sometimes/often/always);
- Frequency of using salt during cooking or food preparation (never/rarely/sometimes/often/always);
- Frequency of consuming salty foods such as pickles, popcorn, chips, sausages, and canned foods (never/rarely/sometimes/often/always);
- Perceived amount of salt consumed (very little/little/average/much/very much);
- Belief that salt or salty sauces in food could lead to health problems (yes/no);
- Perceived importance of reducing salt intake (not important/slightly important/very important).

To enhance interpretability, responses from questions with more than three categories were consolidated into three groups.

The demographic variables including age (categorized as 18–24, 25–39, 40–59, ≥ 60), sex, area of residency (urban/rural), marital status (never married/married/divorced or live separately or widowed), province, and having health insurance (none/basic/basic plus complementary) were used. Education was recorded as the total number of completed school years and categorized into four groups: 0, 1–6, 7–11, and ≥ 12 years. Principal Component Analysis (PCA) was utilized to create the household wealth index using inquiries about essential dwelling attributes and ownership within the research framework. This wealth index was then employed to categorize the population into quintiles, with the first and fifth quintiles representing the least privileged and wealthiest households, respectively (Djalalinia et al. 2022). BMI was defined as underweight (BMI < 18.5 kg/m^2^), normal weight (18.5 ≤ BMI < 25 kg/m^2^), overweight (25 ≤ BMI < 30 kg/m^2^), and obese (BMI ≥ 30 kg/m^2^) for adults (World Health Organization 2025a). Hypertension was defined as having a systolic blood pressure (SBP) ≥ 140 mmHg and/or a diastolic blood pressure (DBP) ≥ 90 mmHg, or currently taking antihypertensive medication (Zhou et al. 2021). Given the established association between salt intake and hypertension, the relationship between salt intake and the cascade of hypertension care was also examined. Hypertension awareness was defined as the proportion of individuals with hypertension who had been previously informed of their condition by a healthcare professional. Hypertension treatment was defined as the proportion of aware hypertensive individuals who were currently taking antihypertensive medications. Hypertension control was defined as the proportion of treated individuals whose SBP was < 140 mmHg and DBP was < 90 mmHg. Strict hypertension control was defined as the proportion of treated individuals with SBP < 120 mmHg and DBP < 80 mmHg. Diabetes was defined as a fasting plasma glucose level ≥ 7.0 mmol/L (126 mg/dL) or current use of medication for elevated blood glucose (Azadnajafabad et al. 2023).

National Cholesterol Education Program ATP III (Adult Treatment Panel III) guideline was applied to assess the total cholesterol (TC), which was classified as desirable (< 200 mg/dL), borderline high (200–239 mg/dL), and high (> 240 mg/dL) (NCEP 2023). The second version of the Global Physical Activity Questionnaire (GPAQ) was used to assess the time spent on physical activities of varying intensity across different domains, including work, transportation, and recreational activities, over a typical week. This information was used to calculate Metabolic Equivalent (MET) minutes per week. Low physical activity was defined as having less than 600 MET minutes per week (World Health Organization 2024). Current smoking was defined as the use of any tobacco product within the past 12 months. Appropriate fruit and vegetable consumption was defined as consuming an average of at least two servings of fruit and three servings of vegetables per day, respectively. Participants were asked whether they had received recommendation on decreasing salt consumption from a physician or any other healthcare professional in the past 12 months.

### 2.4 Statistical Analysis

Data weighting played a crucial role in bridging the gap between data cleaning and analysis. It was essential for addressing incomplete data, non‐response, and disparities in population characteristics. To uphold the reliability and validity of the findings, a weighting procedure was implemented, adjusting the survey data based on various factors. The weighting process comprised four stages: (a) addressing general non‐response, (b) handling non‐response at each survey stage, (c) adjusting for age, sex, and area of residence, and (d) final weighting for data analysis (Djalalinia et al. 2022). Participants were selected through a systematic proportional sampling scheme stratified by province and urban/rural setting. This procedure ensured that the final weighted estimates closely reflected the demographic structure of the Iranian adult population. The representativeness of the weighted sample was validated against the national age and sex distribution, as reported elsewhere (Djalalinia et al. 2022). Weighted frequency, proportion, mean, and standard deviation (SD) were used to describe the data. 95% confidence interval (95% CI) for each quantitative variable was reported. Categorical variables were analyzed by Chi‐squared test. To analyze differences in means between two groups and among three or more groups, an independent‐sample t‐test and the regTermTest function from the “survey” package in R were used, respectively. We used the National Population and Housing Census 2016 conducted by Iran's Statistical Center as the standard population for direct age standardization to compare provinces (GHDx 2025).

To determine the association of various variables with high salt intake (≥ 5 g/day), different logistic regression models were employed. First, univariate logistic regression analyses were conducted to examine the individual association of each variable with the outcome, providing crude odds ratios (ORs) along with 95% confidence intervals (CIs). Subsequently, multivariate models adjusted for age and sex were developed. Final multivariate models included adjustments for sex, BMI categories, appropriate vegetable consumption, low physical activity, and hypertension. These variables were selected based on their significance in the univariate analysis (*p* < 0.05) and clinical plausibility. We limited the number of adjustments in the final model to avoid problems due to the imbalance in the outcome variable.

All data analyses were conducted using R statistical package version 4.3.1. *p*‐value less than 0.05 was considered statistically significant.

### 2.5 Ethical Considerations

The study methodology conformed to Helsinki Declaration standards as revised in 1989. The study was approved by the National Institute for Health Research under the reference code of IR.TUMS.NIHR.REC.1398.006. Participation in this study was voluntary and each participant could leave the study at any time. The aim of the study and the process were explained to all participants, and all provided informed consent before participation in the study in written form. Also, the study design, data collection, data analysis, and paper submission were not affected by the funding source of the study.

## 3 Results

### 3.1 Sociodemographic and Health Characteristics of Participants

For the analyses in this study, 17,910 participants were included in the analysis of salt intake–related outcomes, while 27,838 participants were included in the analysis of outcomes related to attitudes toward salt consumption. Among the included participants, approximately 55% were female, and the majority were in the 40%–59% age group (43.5%), resided in urban areas (75%), and had at least 12 years of schooling (40%). Detailed baseline characteristics of the included participants are presented in Table S1.

### 3.2 Salt Intake

3.2.1

In 2021, salt intake among Iranian adults was substantially above the WHO‐recommended threshold, with 97.98% (97.68%–98.24%) of participants exceeding 5 g/day. The mean salt intake among the Iranian population, based on the Tanaka method, was 9.71 g/day (9.66–9.76). The mean urine sodium and creatinine concentration was 134.35 mmol/L (95% CI: 133.10–135.59) and 146.34 mg/dL (144.70–147.98), respectively (Table S2). Men (9.95 g/day, 9.87–10.3), residents of rural regions (10.2 g/day, 9.94–10.09), people with lower educational levels (9.99 g/day, 9.88–10.01), and married people (9.77 g/day, 9.72–9.83) had higher levels of daily salt intake. Participants aged 25–39 years and 40–59 years showed the lowest and the highest levels of salt intake among various age groups: 9.42 g/day (9.34–9.51) versus 9.86 g/day (9.78–9.93). Smokers consumed lower amounts of salt than non‐smokers: 9.45 g/day (9.31–9.58) versus 9.75 g/day (9.69–9.76) (Table 2).

**TABLE 2:** Mean salt intake and prevalence of inappropriate salt intake in Iran in 2021 by sociodemographic, behavioral, and chronic disease variables.

Category	Mean salt intake (g/day) (95% CI)	*p*	Salt consuming ≥ 5 g/day prevalence (95% CI)	*p*
Total	9.71 (9.66–9.76)	—	97.98 (97.68–98.24)	—
Age groups				
25–39	9.42 (9.34–9.51)	< 0.0001	97.9 (97.4–98.3)	0.243
40–59	9.86 (9.78–9.93)		98.24 (97.84–98.58)	
≥ 60	9.82 (9.71–9.94)		97.61 (96.72–98.26)	
Sex				
Female	9.51 (9.44–9.58)	< 0.0001	97.68 (97.29–98.01)	0.021
Male	9.95 (9.87–10.03)		98.35 (97.84–98.75)	
Area of residency				
Rural	10.02 (9.94–10.09)	< 0.0001	98.1 (97.65–98.46)	0.557
Urban	9.6 (9.54–9.67)		97.94 (97.56–98.26)	
Employment status				
Unemployed	9.43 (9.2–9.66)	< 0.0001	97.49 (95.91–98.47)	0.572
Employed	9.87 (9.79–9.96)		98.19 (97.71–98.57)	
Unpaid work	9.59 (9.52–9.67)		97.85 (97.45–98.19)	
Retired	9.75 (9.57–9.93)		97.99 (96.01–99)	
Insurance				
No insurance	9.69 (9.5–9.89)	0.11	98.69 (97.91–99.19)	0.044
Basic	9.75 (9.69–9.81)		98.1 (97.75–98.39)	
Basic + Complementary	9.62 (9.52–9.72)		97.52 (96.75–98.12)	
Education level (years of schooling)				
0	9.99 (9.88–10.1)	< 0.0001	98.03 (97.4–98.51)	0.327
1–6	9.99 (9.89–10.09)		98.27 (97.77–98.66)	
7–11	9.77 (9.66–9.88)		98.25 (97.63–98.71)	
≥ 12	9.4 (9.31–9.49)		97.65 (97.02–98.14)	
Marriage status				
Single	9.17 (9–9.35)	< 0.0001	96.99 (95.49–98)	0.044
Married	9.77 (9.72–9.83)		98.16 (97.83–98.44)	
Divorced/widow	9.6 (9.43–9.78)		97.28 (96.26–98.03)	
Wealth index				
1 (poorest)	9.78 (9.66–9.9)	0.0001	97.68 (96.99–98.22)	0.296
2	9.61 (9.49–9.72)		97.86 (97.13–98.41)	
3	9.92 (9.82–10.02)		98.39 (97.89–98.77)	
4	9.66 (9.56–9.77)		98.27 (97.64–98.73)	
5 (wealthiest)	9.61 (9.47–9.75)		97.79 (96.69–98.53)	
BMI categories				
Under weight	8.72 (8.43–9.01)	< 0.0001	95.75 (92.97–97.46)	0.0006
Normal	9.27 (9.18–9.36)		97.23 (96.46–97.84)	
Overweight	9.79 (9.71–9.87)		98.27 (97.85–98.61)	
Obese	10.16 (10.06–10.26)		98.61 (98.21–98.92)	
Appropriate fruit consumption				
No	9.74 (9.68–9.81)	0.106	97.85 (97.43–98.2)	0.226
Yes	9.65 (9.57–9.74)		98.19 (97.75–98.54)	
Appropriate vegetable consumption				
No	9.73 (9.68–9.79)	0.0006	98.08 (97.76–98.35)	0.026
Yes	9.43 (9.26–9.6)		96.89 (95.71–97.75)	
Low physical activity				
No	9.71 (9.64–9.78)	0.148	98.33 (97.95–98.64)	0.013
Yes	9.63 (9.55–9.71)		97.57 (97.03–98.02)	
Current smoking				
No	9.75 (9.69–9.81)	< 0.0001	98.03 (97.73–98.29)	0.516
Yes	9.45 (9.31–9.58)		97.66 (96.3–98.53)	
Diabetes				
No	9.7 (9.65–9.76)	0.737	98.12 (97.83–98.37)	0.099
Yes	9.73 (9.58–9.88)		97.13 (95.75–98.07)	
Cholesterol status				
Desirable	9.67 (9.61–9.73)	0.046	97.98 (97.65–98.27)	0.995
Borderline high	9.83 (9.7–9.96)		97.94 (96.99–98.6)	
High	9.87 (9.56–10.17)		97.96 (96.55–98.81)	
Hypertension				
No	9.64 (9.58–9.7)	0.001	98.4 (98.1–98.65)	0.0006
Yes	9.83 (9.73–9.93)		97.21 (96.52–97.77)	
Hypertension awareness				
No	10.11 (9.95–10.27)	< 0.0001	98.73 (97.89–99.24)	< 0.0001
Yes	9.65 (9.53–9.77)		96.25 (95.21–97.07)	
Hypertension treatment				
No	9.79 (9.48–10.11)	0.353	97.53 (94.05–99)	0.231
Yes	9.63 (9.5–9.76)		96.05 (94.91–96.94)	
Hypertension control (SBP < 140 and DBP < 90)				
No	9.74 (9.58–9.9)	0.052	96.32 (94.51–97.55)	0.5
Yes	9.48 (9.26–9.69)		95.66 (94.25–96.73)	
Hypertension strict control (SBP < 120 and DBP < 80)				
No	9.67 (9.54–9.79)	0.286	96.46 (95.25–97.37)	0.02
Yes	9.28 (8.58–9.98)		91.95 (87.44–94.94)	
Receiving decreasing salt recommendation				
No	9.64 (9.54–9.73)	0.092	98.19 (97.72–98.56)	0.293
Yes	9.73 (9.67–9.79)		97.89 (97.5–98.22)	

Marked disparities in salt consumption were observed, with higher intake among participants with obesity or hypertension. People who were underweight had the lowest amount of salt intake, while those with obesity had the highest: 8.72 g/day (95% CI: 8.43–9.01) versus 10.16 g/day (10.06–10.26). Salt intake was higher among patients with hypertension at 9.83 g/day (9.73–9.93) versus 9.64 g/day (9.58–9.7). Among participants with hypertension, those who were aware of their condition consumed lower amounts of salt (9.65 g/day [9.53–9.77] vs. 10.11 g/day [9.95–10.27]) (Table 2). Individuals who added salt to their last meal consumed more salt overall (9.91 g/day [9.82–9.99]) compared to those who did not (9.59 g/day [9.52–9.65]). Participants who considered reducing salt intake “very important” had the lowest mean intake 9.64 g/day (9.57–9.71) (Table 3).

**TABLE 3:** Mean salt intake and prevalence of inappropriate salt intake in Iran in 2021 by variables related to attitudes toward salt consumption.

Category	Salt intake Mean (g/day) (95% CI)	*p*	Salt consuming ≥ 5 g/day prevalence (95% CI)	*p*
Did you use salt in your last lunch or dinner meal?				
No	9.59 (9.52–9.65)	< 0.0001	97.54 (97.09–97.92)	< 0.0001
Yes	9.91 (9.82–9.99)		98.69 (98.35–98.96)	
How often do you add salt to your food while eating or just before meals?				
Never	9.63 (9.54–9.72)	0.001	97.51 (96.96–97.96)	0.007
Rarely/Sometimes	9.68 (9.6–9.76)		98.04 (97.51–98.45)	
Often/Always	9.88 (9.77–9.98)		98.57 (98.06–98.96)	
How often do you use salt when cooking or preparing food?				
Never	9.81 (9.59–10.02)	0.004	97.9 (96.76–98.65)	0.046
Rarely/Sometimes	9.57 (9.47–9.67)		97.34 (96.55–97.95)	
Often/Always	9.76 (9.7–9.82)		98.27 (97.96–98.54)	
How often do you eat salty foods (pickles, popcorn, chips, sausages, and canned foods)?				
Never	9.61 (9.49–9.72)	0.014	96.63 (95.6–97.43)	< 0.0001
Rarely/Sometimes	9.71 (9.65–9.77)		98.22 (97.9–98.49)	
Often/Always	9.86 (9.74–9.99)		99 (98.48–99.35)	
How much salt do you think you consume?				
Very little/Little	9.58 (9.49–9.66)	0.0001	97.45 (96.77–97.98)	0.05
Average	9.74 (9.67–9.81)		98.25 (97.89–98.54)	
Much/Very much	9.95 (9.8–10.1)		98.35 (97.61–98.87)	
Do you think that too much salt or salty sauce in your diet could cause a health problem?				
No	9.63 (9.45–9.82)	0.408	97.49 (94.8–98.8)	0.58
Yes	9.71 (9.66–9.77)		98.02 (97.74–98.26)	
How much important is it to reduce salt?				
Not important	9.95 (9.7–10.2)	0.005	96.84 (95.28–97.89)	0.017
Slightly important	9.78 (9.7–9.87)		98.42 (97.98–98.77)	
Very important	9.64 (9.57–9.71)		97.82 (97.38–98.19)	

Across all respondents, participants who used salt in their most recent meal had 1.77 increased odds of inappropriate salt intake (1.31–2.41). Men had 1.48 (1.04–2.1) times higher odds of consuming inappropriate amounts of salt than women. Compared to the individuals with normal BMI, overweight and obese participants had significantly higher odds of excessive salt intake with adjusted odds ratios of 1.67 (1.2–2.34) and 2.26 (1.57–3.23), respectively. People who had hypertension had lower odds of high salt intake (adjusted OR = 0.48, 0.35–0.66) and the ones who were aware of their condition had even lower odds of high salt intake (adjusted OR = 0.33, 0.17–0.61) (Tables 4 and 5).

**TABLE 4:** Crude and adjusted odds ratios for inappropriate salt intake (≥ 5 g/day) in Iran in 2021, by sociodemographic, behavioral, and chronic disease variables.

Variable	Category	Crude OR	*p*	Age sex adjusted OR	*p*	Adjusted OR^a^	*p*
Age groups	25–39	—	—	—	—	—	—
	40–59	1.2 (0.89–1.63)	0.236	1.2 (0.89–1.63)	0.232	1.27 (0.92–1.75)	0.147
	≥ 60	0.88 (0.59–1.29)	0.505	0.86 (0.58–1.27)	0.448	1.25 (0.86–1.83)	0.239
Sex	Female	—	—	—	—	—	—
	Male	1.42 (1.03–1.96)	0.031	1.44 (1.05–1.97)	0.025	1.48 (1.04–2.1)	0.031
Area of residency	Rural	—	—	—	—	—	—
	Urban	0.92 (0.7–1.22)	0.56	0.93 (0.7–1.22)	0.584	0.92 (0.68–1.23)	0.562
Employment status	Unemployed	—	—	—	—	—	—
	Employed	1.4 (0.8–2.44)	0.243	1.26 (0.71–2.24)	0.429	0.94 (0.51–1.75)	0.852
	Unpaid work	1.17 (0.69–2)	0.561	1.79 (0.99–3.23)	0.055	1.4 (0.73–2.66)	0.31
	Retired	1.26 (0.53–2.99)	0.607	1.26 (0.54–2.93)	0.587	1.04 (0.42–2.6)	0.935
Insurance	No insurance	—	—	—	—	—	—
	Basic	0.68 (0.41–1.13)	0.139	0.69 (0.42–1.14)	0.15	0.76 (0.45–1.29)	0.309
	Basic + Complementary	0.52 (0.3–0.91)	0.021	0.53 (0.31–0.91)	0.021	0.59 (0.33–1.04)	0.066
Education level (years of schooling)	0	—	—	—	—	—	—
	1–6	1.14 (0.78–1.68)	0.495	0.95 (0.63–1.43)	0.798	0.85 (0.56–1.29)	0.445
	7–11	1.13 (0.74–1.71)	0.576	0.83 (0.51–1.34)	0.44	0.77 (0.49–1.21)	0.257
	≥ 12	0.84 (0.58–1.21)	0.343	0.63 (0.37–1.05)	0.078	0.53 (0.34–0.84)	0.006
Marriage status	Single	—	—	—	—	—	—
	Married	1.65 (1.05–2.6)	0.03	1.71 (1.05–2.8)	0.031	1.82 (1.13–2.93)	0.013
	Divorced/widow	1.11 (0.65–1.89)	0.701	1.41 (0.76–2.6)	0.273	1.84 (1.03–3.29)	0.039
Wealth index	1 (poorest)	—	—	—	—	—	—
	2	1.08 (0.73–1.62)	0.692	1.07 (0.71–1.6)	0.745	0.88 (0.57–1.35)	0.555
	3	1.45 (0.99–2.13)	0.057	1.37 (0.93–2.03)	0.114	1.1 (0.73–1.66)	0.655
	4	1.35 (0.89–2.04)	0.157	1.27 (0.83–1.95)	0.271	1.08 (0.69–1.7)	0.727
	5 (wealthiest)	1.05 (0.64–1.72)	0.841	0.98 (0.58–1.65)	0.941	0.81 (0.47–1.42)	0.465
Appropriate fruit consumption	No	—	—	—	—	—	—
	Yes	1.19 (0.89–1.59)	0.23	1.2 (0.9–1.6)	0.217	1.18 (0.86–1.63)	0.302
Appropriate vegetable consumption	No	—	—	—	—	—	—
	Yes	0.61 (0.42–0.88)	0.009	0.62 (0.43–0.89)	0.01	0.6 (0.41–0.88)	0.008
Low physical activity	No	—	—	—	—	—	—
	Yes	0.68 (0.51–0.91)	0.011	0.72 (0.53–0.98)	0.04	0.73 (0.54–1)	0.051
Current smoking	No	—	—	—	—	—	—
	Yes	0.84 (0.51–1.37)	0.486	0.68 (0.41–1.11)	0.124	0.66 (0.39–1.1)	0.109
BMI categories	Normal	—	—	—	—	—	—
	Underweight	0.64 (0.36–1.16)	0.14	0.65 (0.36–1.17)	0.151	0.54 (0.29–0.98)	0.043
	Overweight	1.62 (1.15–2.27)	0.005	1.67 (1.2–2.34)	0.003	1.85 (1.29–2.65)	0.001
	Obesity	2.02 (1.41–2.9)	< 0.0001	2.26 (1.57–3.23)	< 0.0001	2.66 (1.79–3.97)	< 0.0001
Hypertension	No	—	—	—	—	—	—
	Yes	0.57 (0.43–0.76)	< 0.0001	0.52 (0.4–0.67)	< 0.0001	0.48 (0.35–0.66)^b^	< 0.0001
Hypertension awareness	No	—	—	—	—	—	—
	Yes	0.33 (0.18–0.59)	< 0.0001	0.35 (0.2–0.61)	< 0.0001	0.33 (0.17–0.61)^b^	0.001
Hypertension treatment	No	—	—	—	—	—	—
	Yes	0.62 (0.24–1.59)	0.317	0.67 (0.23–1.93)	0.46	0.72 (0.27–1.92)^b^	0.507
Hypertension strict control (SBP < 120 and DBP < 80)	No	—	—	—	—	—	—
	Yes	0.42 (0.23–0.75)	0.003	0.41 (0.23–0.75)	0.004	0.45 (0.25–0.83)^b^	0.01
Hypertension control (SBP < 140 and DBP < 90)	No	—	—	—	—	—	—
	Yes	0.84 (0.5–1.41)	0.51	0.84 (0.5–1.4)	0.501	0.9 (0.54–1.5)^b^	0.688
Diabetes	No	—	—	—	—	—	—
	Yes	0.65 (0.42–1)	0.051	0.65 (0.42–1.01)	0.053	0.78 (0.49–1.25)	0.303
Cholesterol status	Desirable	—	—	—	—	—	—
	Borderline high	0.98 (0.64–1.49)	0.925	0.98 (0.65–1.5)	0.941	0.94 (0.61–1.44)	0.774
	High	0.99 (0.56–1.74)	0.973	1.03 (0.58–1.83)	0.921	0.93 (0.53–1.65)	0.813
Receiving decreasing salt recommendation	No	—	—	—	—	—	—
	Yes	0.86 (0.64–1.15)	0.301	0.87 (0.65–1.16)	0.339	0.87 (0.63–1.18)	0.359

^a^ Adjusted by sex, BMI categories, appropriate vegetable consumption, low physical activity, and hypertension.

^b^ Adjusted by sex, BMI categories, appropriate vegetable consumption, and low physical activity.

**TABLE 5:** Crude and adjusted odds ratios for inappropriate salt intake (≥ 5 g/day) in Iran in 2021, variables related to attitudes toward salt consumption.

Variable	Category	Crude OR	*p*	Age sex adjusted OR	*p*	Adjusted OR^a^	*p*
Did you use salt in your last lunch or dinner meal?	No (ref)	—	—	—	—	—	—
	Yes	1.9 (1.42–2.54)	< 0.0001	1.84 (1.37–2.46)	< 0.0001	1.77 (1.31–2.41)	< 0.0001
How often do you add salt to your food while eating or just before meals?	Never (ref)	—	—	—	—	—	—
	Rarely/Sometimes	1.28 (0.93–1.75)	0.132	1.24 (0.9–1.71)	0.195	1.18 (0.84–1.66)	0.329
	Often/Always	1.77 (1.22–2.57)	0.003	1.69 (1.16–2.48)	0.007	1.6 (1.07–2.4)	0.021
How often do you use salt when cooking or preparing food?	Never (ref)	—	—	—	—	—	—
	Rarely/Sometimes	0.78 (0.47–1.32)	0.361	0.81 (0.48–1.39)	0.448	0.71 (0.41–1.26)	0.241
	Often/Always	1.22 (0.76–1.97)	0.415	1.3 (0.8–2.12)	0.289	1.17 (0.7–1.98)	0.548
How often do you eat salty foods (pickles, popcorn, chips, sausages, and canned foods)?	Never (ref)	—	—	—	—	—	—
	Rarely/Sometimes	1.92 (1.39–2.67)	< 0.0001	1.96 (1.41–2.73)	< 0.0001	1.69 (1.22–2.33)	0.002
	Often/Always	3.47 (2.08–5.77)	< 0.0001	3.54 (2.11–5.95)	< 0.0001	2.74 (1.64–4.59)	< 0.0001
How much salt do you think you consume?	Very little/Little (ref)	—	—	—	—	—	—
	Average	1.47 (1.08–1.99)	0.014	1.49 (1.12–1.98)	0.007	1.41 (1.04–1.92)	0.028
	Much/Very much	1.57 (1–2.46)	0.051	1.52 (0.98–2.36)	0.065	1.52 (0.95–2.44)	0.081
Do you think that too much salt or salty sauce in your diet could cause a health problem?	No (ref)	—	—	—	—	—	—
	Yes	1.27 (0.59–2.74)	0.537	1.29 (0.61–2.73)	0.503	1.24 (0.57–2.73)	0.587
How much important is it to reduce salt?	Not important (ref)	—	—	—	—	—	—
	Slightly important	2.04 (1.25–3.32)	0.004	2.12 (1.3–3.45)	0.003	1.6 (0.94–2.73)	0.083
	Very important	1.47 (0.93–2.32)	0.1	1.57 (0.98–2.5)	0.06	1.3 (0.77–2.17)	0.324

^a^ Adjusted by sex, BMI categories, appropriate vegetable consumption, low physical activity, and hypertension.

**FIGURE 1:**
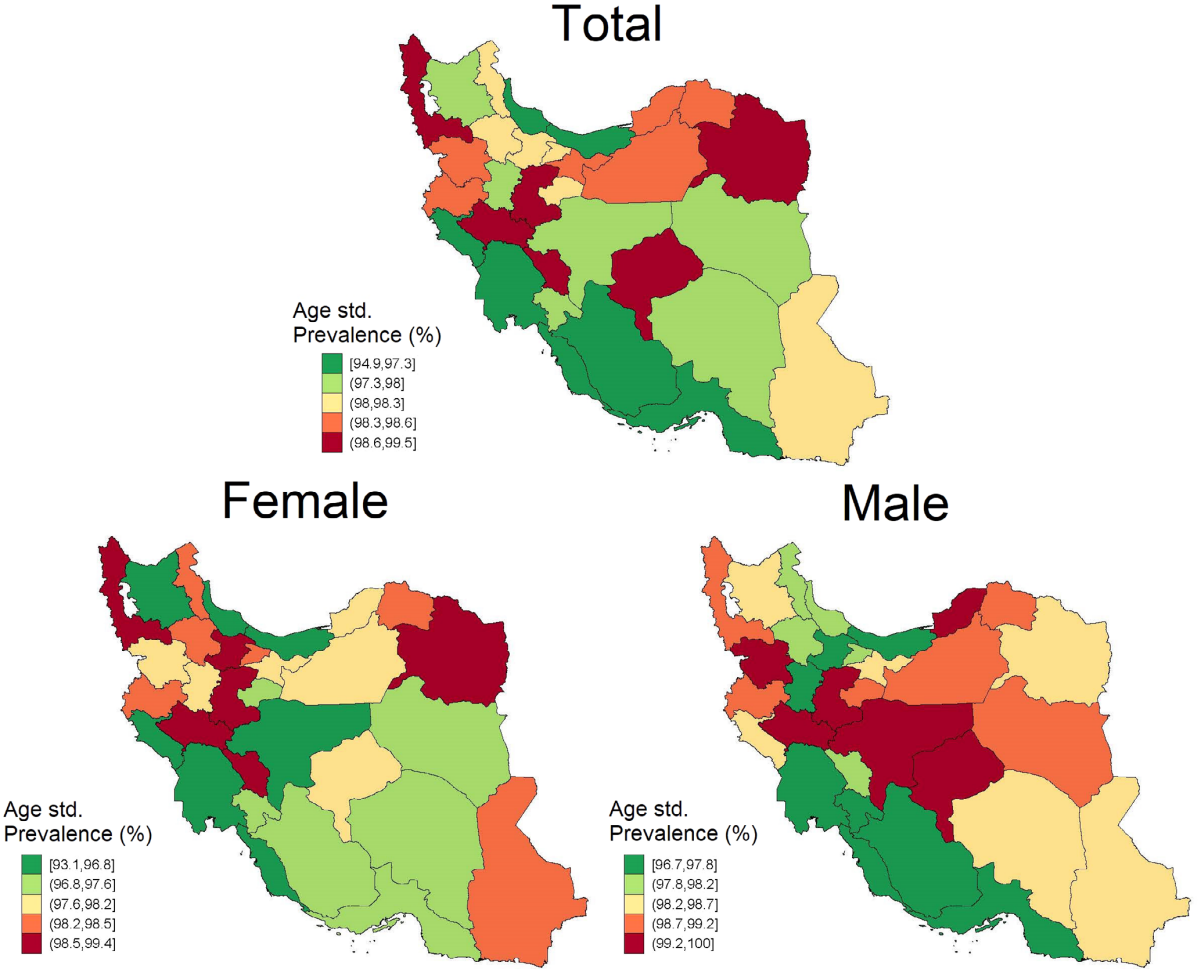
High salt intake prevalence in different provinces of Iran.

There was no association between hypertension treatment or hypertension control and inappropriate salt consumption. However, individuals whose hypertension was strictly controlled had lower odds of consuming salt inappropriately (adjusted OR = 0.45, 0.25–0.83). In an age‐standardized model for the sub‐national level, Markazi had the highest salt intake prevalence (99.47%) and Bushehr had the lowest salt intake (94.92%) in both sexes (Figure 1).

**FIGURE 2:**
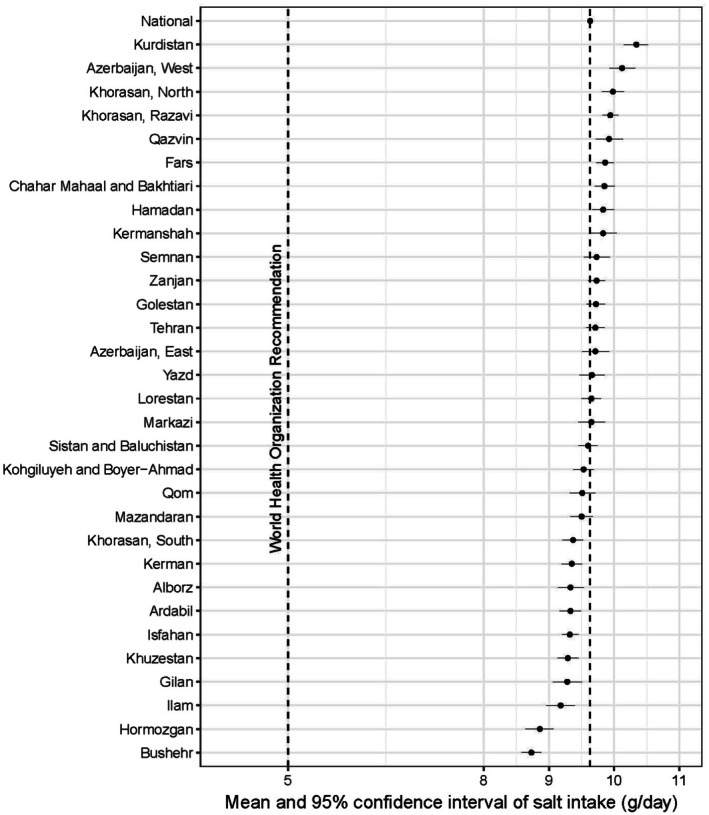
Mean and 95% confidence interval of salt intake (g/day) in all provinces of Iran.

Although salt intake in all provinces was higher than the WHO recommended amount, some variations were observable, from 10.34 g/day in Kurdistan, the province with the highest, to 8.73 g/day in Bushehr, the province with the lowest amount of salt intake (Figure 2).

### 3.3 Attitudes Toward Salt Intake

3.3.1

Although the majority of participants recognized the health risks of excessive salt, there remained a considerable gap between knowledge and behavior, with more than one‐third still adding salt at their last meal. Overall, 91.96% (91.59%–92.31%) of participants thought that too much salt in their diet could cause health problems and 57.93% (57.28%–58.58%) of the participants thought that it is “very important” to reduce salt intake. 38.22% (37.59%–38.86%) of the individuals used salt in their last meal. 23.99% (23.17%–24.83%) of men “often/always” added salt just before or while eating a meal, which was higher than women (21.54% [20.83%–22.27%]). Younger adults (age 25–39) reported the highest frequency of adding salt to their last meal 42.22% (41.05%–43.4%), followed by the 40–59 age group 38.79% (37.77%–39.82%). In contrast, only 30.5% (29.21%–31.82%) of those aged ≥ 60 reported this behavior, indicating a decline in salt‐adding habit with increasing age. 70.38% (68.98%–71.74%) of people who were aware of their hypertension status believed that it is “very important” to reduce salt intake (Table S3).

## 4 Discussion

The findings of our study underscore the alarming prevalence of excessive salt intake among the Iranian adult population, nearly doubling the WHO recommendations. While the mean salt intake showed a slight increase from 9.52 g/day (95% CI: 9.48–9.56) in 2016 to 9.71 g/day (9.66–9.76) in 2021 (Rezaei et al. 2018), this change falls within the margin of measurement error and may not represent a meaningful upward trend. Nonetheless, the consistently high intake across both time points is a cause for concern. Furthermore, disparities persist across sociodemographic groups, with men, rural residents, and married individuals continuing to exhibit higher levels of salt consumption. Additionally, age disparities were evident, with individuals aged 40–59 demonstrating the highest salt intake, while those aged 25–39 exhibited the lowest levels.

There was an association between salt intake and certain health conditions, namely obesity and hypertension. Participants with unhealthy lifestyle habits, such as using saltshakers, adding salt during meals, inadequate vegetable consumption, and low physical activity, exhibited higher salt intake levels. Considering the different components of the hypertension cascade of care, only awareness and strict control were directly associated with appropriate salt intake. As this study is cross‐sectional, causal inferences cannot be made; the lower salt intake among hypertensive individuals, with adjusted OR of 0.48 for inappropriate salt consumption, likely reflects reverse causality, which means individuals who are aware of their hypertension may intentionally reduce their salt intake.

Over the years, there has been a concerning increase in the mean salt intake among the Iranian population, indicating a persistent challenge in salt reduction efforts (Rezaei et al. 2018). Interestingly, our study found that individuals who added salt to their meals exhibited significantly higher salt intake levels, suggesting a potential avenue for intervention by discouraging the use of saltshakers or advocating for reduced‐holed shakers. In this sense, the design of saltshakers could also play a role in reducing salt intake. For instance, a study showed that using five‐holed saltshakers, in contrast to 17‐holed ones, was associated with exhibiting lower salt intake per meal. This suggests that not only refraining from using salt shakers but also selecting designs with fewer holes may contribute to reducing overall salt intake (Goffe et al. 2016).

The elderly and older adults demonstrated higher salt intake, which could be due to perceiving lower taste intensity compared with young adults (Sato et al. 2022). Sex‐based differences in salt intake were also evident, with men consuming more salt than women. This aligns with previous research indicating similar sex disparities in salt intake, even among children, and across rural and urban settings. Such global patterns emphasize the importance of considering sex‐specific approaches in salt reduction initiatives (Emamian et al. 2021).

Traditional bread emerged as a significant contributor to salt intake in the Iranian diet, highlighting the need for interventions in bread production, such as salt substitutes and quality improvements in flour. Challenges in implementing these measures include limited competition in flour or bread usage. Nevertheless, enhancing bakery supervision, especially in traditional bakeries, and imposing penalties for non‐compliance could advance salt reduction initiatives (Loloei et al. 2019; Hadian et al. 2022). There might be a relationship between low‐quality flour used in rural areas and higher amounts of salt intake in residents of rural regions, which needs further investigation in future studies. International comparisons reveal varying salt intake levels across countries, with factors influencing reductions remaining unclear (Thout et al. 2019). Efforts to promote salt avoidance while cooking and implement reduction strategies in bakeries align with initiatives observed in other countries, such as Turkey (Erdem et al. 2017, 2010) and Japan (Imamoto et al. 2021).

Our study reinforces the importance of education in promoting healthy eating habits, particularly among individuals with obesity or hypertension. Encouragingly, well‐informed patients with hypertension demonstrated significant changes in salt intake habits, highlighting the potential impact of targeted education initiatives (Mohammadifard et al. 2019). However, there remains a gap between knowledge and action, as evidenced by participants' underestimation of their daily salt intake (Leyvraz et al. 2018). For instance, a study on older rural Chinese adults revealed that they often recognized the health risks of excessive salt yet continued high consumption due to entrenched dietary habits and cultural practices (Zhang et al. 2016). Likewise, a recent study in Polish children and adolescents showed that reductions in salt intake were strongly shaped by socio‐environmental factors such as the home food environment, peer influence, and school education policies (Malczyk et al. 2024).

Our findings highlight several policy priorities for reducing salt intake in Iran. Reformulating bread and packaged foods to lower salt content, coupled with front‐of‐package labeling and stricter bakery regulation, should be central strategies. In addition, discouraging salt shaker use and delivering culturally tailored education campaigns through schools, health centers, and media can help translate awareness into healthier behaviors.

Globally, efforts to reduce salt intake have gained momentum, with various strategies implemented across countries. These include food reformulation, consumer education (Djalalinia et al. 2022), food labeling (Toora and Rajagopal 2002), public institution interventions, and taxation, all aimed at driving behavioral change and lowering daily salt intake (Webster et al. 2011; Jawaldeh et al. 2018). To achieve the WHO's recommended daily salt intake limits, comprehensive strategies outlined in the SHAKE package are essential (World Health Organization 2023a). These include surveillance, industry collaboration, standards for labeling and marketing, knowledge dissemination, and environmental interventions. Additionally, strategies elaborated by Resolve to Save Lives emphasize the importance of government support, industry cooperation, and social media engagement in driving salt reduction efforts (Resolve to Save Lives 2025). These strategies encompass six steps. Firstly, educating the public about the advantages of behavioral modifications has proven successful in Australia, leading to a 10% reduction in salt intake (Land et al. 2016). Secondly, promoting the use of low‐sodium salt, incorporating potassium chloride as a substitute for sodium chloride, has shown effectiveness in China (Shao et al. 2017). Thirdly, introducing front‐of‐package labeling to highlight high‐salt content has yielded positive outcomes, as demonstrated by Chile's mandatory labeling program preventing schools from purchasing products with warning labels (Reyes et al. 2019). Fourthly, enforcing specific salt reduction targets on food industries has been successful, as seen in Kuwait, where a major bread manufacturer achieved a 20% reduction in salt content (Alhamad et al. 2015). Fifthly, implementing salt reduction standards in various settings such as hospitals, worksites, and schools, following successful models in the United Kingdom, Australia, and the United States, has proven beneficial (Micha et al. 2018). Lastly, reducing salt content in foods prepared outside the home, particularly in restaurants, has been effective in certain chain restaurants in the United States, which were compelled to include detailed nutrition information, including salt content, on their menus (Resolve to Save Lives 2022).

Excessive salt intake has been linked to various health conditions, including cardiovascular diseases, stroke, and kidney disorders (Chen et al. 2021). The economic burden associated with treating these conditions further underscores the urgency of implementing effective salt reduction strategies. Moreover, the impact of high salt intake extends beyond individual health outcomes, affecting healthcare systems, productivity, and overall societal well‐being (GBD 2017 Diet Collaborators 2019).

One area that warrants attention is the role of cultural factors in shaping dietary habits and salt intake patterns. Cultural preferences and traditions often influence food choices, cooking methods, and meal preparation techniques. In the Iranian context, traditional dishes and culinary practices may contribute significantly to salt intake, highlighting the importance of culturally sensitive approaches to salt reduction. Engaging communities, religious leaders, and culinary experts in awareness campaigns and educational initiatives can facilitate the adoption of healthier dietary practices while respecting cultural norms (Amerzadeh et al. 2022).

One of the most effective strategies to reduce salt intake is the use of potassium‐enriched salt substitutes (K‐LSS). Large randomized controlled trials have demonstrated that K‐LSS can lower blood pressure and reduce cardiovascular events without increasing the risk of clinical hyperkalemia, arrhythmia, or sudden death, despite a slight rise in mean serum potassium (Neal et al. 2021). Consistent with current hypertension guidelines (Writing Committee Members et al. 2025), K‐LSS should be recommended for most patients, with serum potassium monitoring reserved for individuals with chronic kidney disease or those taking potassium‐sparing medications (Yuan et al. 2023).

Moreover, integrating salt reduction efforts into broader public health agendas, such as campaigns targeting non‐communicable diseases and promoting healthy lifestyles, can maximize impact and sustainability (Ma et al. 2017). By aligning salt reduction initiatives with existing health promotion strategies, policymakers can leverage resources, infrastructure, and public support to drive meaningful change. Addressing the complex challenge of excessive salt intake requires a multifaceted approach that considers sociocultural, environmental, and behavioral factors. In summary, reducing excessive salt intake is not only a national priority for Iran but also a global public health imperative. Achieving this goal will require coordinated action across sectors, combining food reformulation, culturally sensitive education, community engagement, and stronger regulatory frameworks to create environments that support healthier dietary choices and ultimately reduce the burden of salt‐related diseases.

## 5 Strengths and Limitations

This study benefits from a large and nationally representative sample with a balanced sex ratio, encompassing both men and women. The sampling design ensured representativeness at both the national and subnational levels, covering all 31 provinces of Iran and capturing both urban and rural populations. In addition, electronic data collection tools with built‐in validation and consistency checks were used, which enhanced data accuracy, minimized missing values, and improved data quality. Field teams were extensively trained, and data collection procedures were standardized and closely supervised to ensure uniform implementation across provinces. Furthermore, the methodological framework followed the WHO STEPS protocol, allowing comparability with previous rounds of the survey and enabling monitoring of national trends and progress over time.

However, our study also has its limitations. Firstly, laboratory data collection was restricted to adults aged 25 years and older, thus lacking data from children, teenagers, and younger adults, which may limit the generalizability of biochemical estimates. Secondly, sodium intake was estimated from a single spot urine using published prediction equations. Although convenient, single‐spot approaches exhibit substantial error at the individual level and can misclassify persons around intake thresholds due to diurnal variation, hydration effects, and model bias; therefore, they are unsuitable for individual‐level inference (Allen et al. 2017). Consistent with surveillance guidance and comparative evaluations, spot‐urine methods are more appropriate for estimating population mean intake, where bias is smaller relative to 24‐h collections (McLean 2014). Applying equations developed in other populations may introduce additional error, and residual within‐person day‐to‐day variability remains (Cogswell et al. 2013). Accordingly, our provincial and national means are emphasized, and any threshold‐based prevalence is interpreted as a population indicator rather than an individual measure (McLean 2014). Future work could incorporate gold‐standard 24‐h collections or repeat‐spot calibration subsamples to refine estimates and reduce misclassification (Cogswell et al. 2013). Thirdly, the cross‐sectional design of the study restricts interpretation to associations; rather than causality, observed associations should therefore be interpreted as descriptive rather than causal. Lastly, the completion of questionnaires relied on self‐reporting, potentially introducing recall bias among participants, which may have led to misleading data.

## 6 Conclusion

In conclusion, our study highlights the concerning prevalence of excessive salt intake among Iranian adults, nearly doubling the WHO recommendations. Unhealthy habits such as using salt shakers, adding salt during cooking or meals, and consuming salty processed foods were more common in individuals with higher salt intake, highlighting behavioral patterns that may be relevant for public health initiatives. Continued attention to culturally informed strategies may help address these behaviors, including discouraging salt shaker use, reformulating bread and processed foods, and promoting healthier cooking practices. Policymakers should prioritize public education, strengthen food industry regulation, and integrate salt reduction into broader NCD prevention agendas. Collaborative efforts among health authorities, legislators, food producers, and community stakeholders are essential to translate awareness into action and achieve meaningful reductions in salt intake.

## Author Contributions

**Nasim Nosratinia:** writing – original draft (equal), writing – review and editing (equal). **Sina Azadnajafabad:** validation (equal), writing – original draft (equal), writing – review and editing (equal). **Masoud Masinaei:** data curation (equal), formal analysis (equal), methodology (equal), validation (equal), visualization (equal), writing – review and editing (supporting). **Ali Golestani:** formal analysis (equal), validation (equal), visualization (equal), writing – original draft (supporting), writing – review and editing (equal). **Seyyed‐Hadi Ghamari:** validation (equal), visualization (equal), writing – original draft (supporting), writing – review and editing (supporting). **Mohsen Abbasi‐Kangevari:** validation (equal), writing – original draft (supporting), writing – review and editing (supporting). **Negar Rezaei:** conceptualization (equal), data curation (equal), investigation (equal), methodology (equal), project administration (equal), resources (equal), supervision (equal), validation (equal), writing – review and editing (supporting). **Sepehr Khosravi:** formal analysis (equal), visualization (equal), writing – review and editing (supporting). **Shahabeddin Rezaei:** methodology (equal), writing – review and editing (supporting). **Naser Ahmadi:** data curation (equal), formal analysis (equal), methodology (equal), validation (equal), visualization (equal), writing – review and editing (supporting). **Ameneh Kazemi:** data curation (equal), writing – review and editing (supporting). **Erfan Ghasemi:** data curation (equal), formal analysis (equal), methodology (equal), validation (equal), visualization (equal), writing – review and editing (supporting). **Yosef Farzi:** data curation (equal), writing – review and editing (supporting). **Mohammad‐Mahdi Rashidi:** data curation (equal), writing – review and editing (supporting). **Moein Yoosefi:** data curation (equal), writing – review and editing (supporting). **Nazila Rezaei:** data curation (equal), writing – review and editing (supporting). **Maryam Nasserinejad:** formal analysis (equal), writing – review and editing (supporting). **Rosa Haghshenas:** data curation (equal), writing – review and editing (supporting). **Sahar Mohammadi Fateh:** data curation (equal), writing – review and editing (supporting). **Mohammad Keykhaei:** writing – review and editing (supporting). **Mana Moghimi:** data curation (equal), writing – review and editing (supporting). **Elmira Foroutan Mehr:** data curation (equal), writing – review and editing (supporting). **Azadeh Momen Nia Rankohi:** writing – review and editing (supporting). **Shirin Djalalinia:** conceptualization (equal), data curation (equal), investigation (equal), methodology (equal), supervision (equal), writing – review and editing (supporting). **Farshad Farzadfar:** conceptualization (lead), data curation (equal), funding acquisition (lead), investigation (equal), methodology (equal), project administration (equal), resources (equal), supervision (equal), validation (equal), writing – review and editing (equal).

## Funding

This research was conducted with the support of the National Institute of Health Research of the I.R. Iran and Tehran University of Medical Sciences (Contract No. 241/M/9839). The funding body had no direct role in any step of study design, data collection, analysis, interpretation, or writing the manuscript.

## Ethics Statement

The study was approved by the National Institute for Health Research under the reference code of IR.TUMS.NIHR.REC.1398.006. Participation in this study was voluntary, and each participant could leave the study at any time. The aim of the study and the process were explained to all participants, and all provided informed consent prior to participation in the study in written form. Also, the study design, data collection, data analysis, and paper submission were not affected by the funding source of the study.

## Consent

The authors have nothing to report.

## Conflicts of Interest

The authors declare no conflicts of interest.

## Supporting information


**Table S1:** fsn371399‐sup‐0001‐TableS1.docx.

**Table S2:** fsn371399‐sup‐0002‐TableS2.docx.

**Table S3:** fsn371399‐sup‐0003‐TableS3.xlsx.

## Data Availability

The datasets used and analyzed during the current study are available from the corresponding author on reasonable request.
