# Supplementary material for: Salt Intake Among the Iranian Population and Public Attitudes Toward Salt Consumption: National and Subnational Report From STEPS 2021
Source: Food Sci Nutr. 2025 Dec 30;14(1):e71399. doi: 10.1002/fsn3.71399 (PMC12750446; doi:10.1002/fsn3.71399)
Supplement: Supplementary file 1 — Table S1: fsn371399‐sup‐0001‐TableS1.docx. [file FSN3-14-e71399-s003.docx]

Supplementary Table 1. Baseline characteristics of included participants in the study

| Variable | Category | Participants included for attitude to salt consumption outcomes from step 1 (number (weighted prevalence%)) | Participants included for salt intake outcomes from step 3 (number (weighted prevalence%)) |
| --- | --- | --- | --- |
| Total | Total | 27838 | 17910 |
| sex | Female | 15369 (55.33) | 10175 (55.58) |
|  | Male | 12469 (44.67) | 7735 (44.42) |
| Age groups | 18-24 | 2669 (9.32) | - |
|  | 25-39 | 8473 (30.12) | 5777 (32.99) |
|  | 40-59 | 10743 (39.09) | 7965 (43.51) |
|  | >= 60 | 5953 (21.47) | 4168 (23.49) |
| Area of residency | Rural | 7587 (25) | 5792 (24.68) |
|  | Urban | 20251 (75) | 12118 (75.32) |
| Education level (years of schooling) | 0 | 4014 (13.49) | 3132 (14.66) |
|  | 1-6 | 6740 (23.78) | 5000 (25.63) |
|  | 7-11 | 5187 (19.09) | 3332 (19.3) |
|  | >= 12 | 11707 (43.64) | 6329 (40.41) |
| Marriage status | Single | 4243 (15.05) | 1399 (8.26) |
|  | Married | 21327 (76.57) | 14919 (82.58) |
|  | Divorced/widow | 2268 (8.37) | 1592 (9.16) |
| Employment status | Unemployed | 1548 (5.27) | 896 (4.57) |
|  | Employed | 10078 (36.95) | 6464 (38.19) |
|  | Unpaid work | 13485 (48.46) | 8723 (46.83) |
|  | Retired | 2537 (9.32) | 1710 (10.41) |
| Wealth index | 1 (poorest) | 5266 (19.04) | 3625 (19.33) |
|  | 2 | 5262 (20.64) | 3295 (20.15) |
|  | 3 | 5261 (18.94) | 3644 (18.94) |
|  | 4 | 5260 (19.98) | 3441 (20.29) |
|  | 5 (wealthiest) | 5258 (21.4) | 2951 (21.29) |
| Insurance | No insurance | 2548 (9.83) | 1370 (8.92) |
|  | Basic | 17402 (61.87) | 11538 (62.11) |
|  | Basic + Complementary | 7573 (28.3) | 4818 (28.97) |
