# Supplementary material for: Salt Intake Among the Iranian Population and Public Attitudes Toward Salt Consumption: National and Subnational Report From STEPS 2021
Source: Food Sci Nutr. 2025 Dec 30;14(1):e71399. doi: 10.1002/fsn3.71399 (PMC12750446; doi:10.1002/fsn3.71399)
Supplement: Supplementary file 2 — Table S2: fsn371399‐sup‐0002‐TableS2.docx. [file FSN3-14-e71399-s002.docx]

| **Supplementary Table 2.** Participant’s laboratory data | | | | |
| --- | --- | --- | --- | --- |
| **Variables** | **Total**  **(Mean, 95% CI)** | **Men**  **(Mean, 95% CI)** | **Women**  **(Mean, 95% CI)** | **p-value** |
| Spot urine sodium (mmol/l) | 134.35,  (133.1-135.59) | 142.15,  (140.28-144.02) | 128.12,  (126.47-129.76) | <0.001 |
| Spot urine creatinine (mg/dl) | 146.34,  (144.7-147.98) | 161.18,  (158.69-163.68) | 134.49,  (132.41-136.57) | <0.001 |
